# Mixture-Amount Design and Response Surface Modeling to Assess the Effects of Flavonoids and Phenolic Acids on Developmental Performance of *Anastrepha ludens*

**DOI:** 10.1007/s10886-014-0404-6

**Published:** 2014-03-12

**Authors:** Carlos Pascacio-Villafán, Stephen Lapointe, Trevor Williams, John Sivinski, Randall Niedz, Martín Aluja

**Affiliations:** 1 Red de Manejo Biorracional de Plagas y Vectores, Instituto de Ecología, A.C. (INECOL), Antigua Carretera a Coatepec No. 351, Xalapa, Veracruz CP 91070 Mexico; 2 United States Horticultural Research Laboratory, Fort Pierce, FL USA; 3 Center for Medical, Agricultural and Veterinary Entomology, Gainesville, FL USA

**Keywords:** *Anastrepha ludens*, Larval performance, Phenolic compounds, Response-surface modeling, Secondary compounds, Tephritidae, Diptera

## Abstract

Host plant resistance to insect attack and expansion of insect pests to novel hosts may to be modulated by phenolic compounds in host plants. Many studies have evaluated the role of phenolics in host plant resistance and the effect of phenolics on herbivore performance, but few studies have tested the joint effect of several compounds. Here, we used mixture-amount experimental design and response surface modeling to study the effects of a variety of phenolic compounds on the development and survival of Mexican fruit fly (*Anastrepha ludens* [Loew]), a notorious polyphagous pest of fruit crops that is likely to expand its distribution range under climate change scenarios. (+)- Catechin, phloridzin, rutin, chlorogenic acid, and *p*-coumaric acid were added individually or in mixtures at different concentrations to a laboratory diet used to rear individuals of *A. ludens*. No effect was observed with any mixture or concentration on percent pupation, pupal weight, adult emergence, or survival from neonate larvae to adults. Larval weight, larval and pupal developmental time, and the prevalence of adult deformities were affected by particular mixtures and concentrations of the compounds tested. We suggest that some combinations/concentrations of phenolic compounds could contribute to the management of *A. ludens*. We also highlight the importance of testing mixtures of plant secondary compounds when exploring their effects upon insect herbivore performance, and we show that mixture-amount design is a useful tool for this type of experiments.

**Electronic supplementary material:**

The online version of this article (doi:10.1007/s10886-014-0404-6) contains supplementary material, which is available to authorized users.

## Introduction

Phenolic compounds occur in all plant vegetative structures, flowers, fruits and seeds (Croteau et al. 2000; Lattanzio et al. 2006). Phenolics such as flavonoids, phenolic acids, coumarins, and tannins appear to play critical roles in ecological interactions required for plant survival (Appel 1993; Lattanzio et al. 2006). For example, flavonoids and phenolic acids deter feeding, suppress larval growth, decrease weight gain, and increase mortality of phytophagous insects in at least four orders (Dowd and Vega 1996; Fulcher et al. 1998; Ikonen et al. 2001; Lindroth and Peterson 1988; Pree 1977; Salvador et al. 2010).

Feeding experiments involving the study of individual phenolic compounds greatly outnumber studies on mixtures. However, because phenolics do not occur in isolation in host plants, it has been suggested that synergistic or antagonistic activities are likely (Calcagno et al. 2002; Gershenzon et al. 2012; Onyilagha et al. 2012). Studies addressing mixtures require a statistical approach based on mixture polynomials developed by Scheffé (Cornell 2002). The method accounts for the mixture constraint in which *x*_*1*_ *, x*_*2*_ *,…, x*_*p*_ are proportions of *p* components of a mixture, such that 0 ≤ *x*_*i*_ ≤ 1, where *i* = 1, 2,…, *p*; and *x*_*1*_ + *x*_*2*_ + *…* + *x*_*p*_ = 1 (*i.e.*, 100 % of the composition of the experimental treatment) (Anderson and Whitcomb 2005; Montgomery 2001). These so-called mixture experiments allow simultaneous examination of multiple components and their interactions, thereby making them particularly useful for modeling synergistic and antagonistic effects (Busch and Phelan 1999; Lapointe et al. 2008). Mixture experiments have been used in engineering, chemical, pharmaceutical, and food industries (Bondari 2005; Dal Bello and Vieira 2011). Strikingly, they have not been widely adopted in ecological research, particularly in diet experiments to study the effects of plant secondary metabolites on phytophagous insects, despite being recognized as a potentially valuable tool for the study of interactions in plant and insect ecology (Beanland et al. 2003; Busch and Phelan 1999; Lapointe et al. 2010; O’Hea et al. 2010).

The Mexican fruit fly, *Anastrepha ludens* (Loew) (Diptera: Tephritidae), is a polyphagous insect with more than 40 known natural host plant species (Norrbom 2004). It is a major pest of fruit crops such as mango (*Mangifera indica* L.), and citrus (*Citrus* spp.), from southern Texas southward through Mexico and Central America (Aluja et al. 1996; Birke et al. 2013). *Anastrepha ludens* is regarded as a potential invader of novel environments, where it could exploit new hosts causing severe disturbance to natural or agricultural ecosystems (Aluja and Mangan 2008; Birke et al. 2013). Recent work suggests that the invasion of this pest fly could be hindered by enhancing the levels of phenolic compounds on potential host fruit (Aluja et al. 2014).

We used a mixture-amount design experiment (Piepel and Cornell 1985) to examine the effects of flavonoids and phenolic acids on the development and survival of diet-reared *A. ludens*. Our study system was based on phenolic compounds found in apples (*Malus* × *domestica* Borkh), a potential host of *A. ludens* under climate change scenarios (Aluja et al. 2014). We predicted that blends of phenolic compounds at high concentrations would affect insect development and survival more than individual compounds at low concentrations.

## Methods and Materials

### Test Compounds

We tested the flavonoids (+)-catechin, phloridzin, and rutin, and the phenolic acids chlorogenic acid and *p*-coumaric acid. Except for *p*-coumaric acid, all compounds tested were found at higher levels in apple cultivars that were resistant to *A. ludens* attack, and at lower levels in those found to be susceptible (Aluja et al. 2014). *p*-Coumaric acid is a common phenolic acid found in apple pulp (Biedrzycka and Amarowicz 2008). Therefore, *A. ludens* would certainly encounter mixtures of these compounds when feeding on apples. In addition, all compounds tested affect the development of tephritids and other phytophagous insects (Fulcher et al. 1998; Pree 1977; Stamp and Osier 1997). All compounds were purchased from Sigma-Aldrich Company (Toluca, Mexico) and differed in their chemical properties (Supplementary Table 1).

### Source of Insects

Larvae of *A. ludens* were obtained from a laboratory colony reared on an artificial diet in the laboratories of the Red de Manejo Biorracional de Plagas y Vectores (RMBPV) at the Instituto de Ecología, A.C. (INECOL), Xalapa, Veracruz State, Mexico (Aluja et al. 2009).

### General Procedure

We worked with the artificial diet commonly used at the RMBPV to rear *A. ludens* for experimental purposes, which is based on dried yeast (9.7 %), wheat germ (9.7 %), sugar (9.7 %), vitamins (0.14 %), corn cob fractions (14.55 %), water (54.85 %), sodium benzoate (0.78 %), and hydrochloric acid (0.58 %) (Aluja et al. 2009). Samples of 25 g of artificial diet were placed in a Petri dish (5 cm diam × 2 cm high) together with 30 *A. ludens* neonate larvae (<6 hr old). Various phenolic compounds were added to the diet, as described in the “*Experimental Approach*” section.

Petri dishes with diet and larvae were placed inside plastic containers (7 cm diam × 6 cm high) containing a 3 cm layer of vermiculite as a pupation substrate. Plastic containers were closed with a lid that had a 5 cm diam hole covered with organdy cloth, and were placed in a dark room at 30 ± 1 °C and 70 ± 5 % RH. Pupation was checked daily beginning 7 d after the start of the experiment. All pupae found in vermiculite were removed and held individually inside clean Petri dishes (4 cm diam × 1.5 cm high) with vermiculite and a perforated lid to allow ventilation. Pupae were incubated for 3 d at 27 ± 1 °C, 63 ± 5 % RH, and photoperiod of 12: 12 (L:D) and then were individually weighed using an analytical balance (Sartorius CP64) and returned to their Petri dishes until adult emergence.

### Response Variables

We assessed the development and survival of *A. ludens* by measuring: 1) larval development time (days), calculated as the mean time that the larval stage lasted in each diet; 2) larval weight (mg), measured by weighing individually all 7-d-old larva from each diet and calculating the mean weight; 3) pupal development time (days), calculated as the mean time that the pupal stage lasted in each diet; 4) pupation (%), expressed as the percentage of larvae molting into pupae in relation to the total of larvae placed in each diet; 5) pupal weight (mg), measured by weighing individually all 3-d-old pupae from each diet and calculating the mean weight; 6) adult emergence, calculated as the percentage of adults that emerged in relation to the total of pupae from each diet; 7) percentage of survival from neonate to adult, calculated as the percentage of emerged flies in relation to the total number of larvae placed on each diet; 8) percentage of deformed adults (those emerged with atrophied wings, atrophied ovipositor or lacked one wing), estimated in relation to the total number of emerged adults.

### Experimental Approach

The study was designed as a mixture-amount experiment, and included five mixture components: (+)-catechin, phloridzin, rutin, chlorogenic acid, and *p*-coumaric acid, and one numerical factor: the total concentration of phenolic compounds in the experimental diet. Because (+)-catechin, phloridzin, rutin, chlorogenic acid, and *p*-coumaric acid were treated as components of a mixture, the range of each component was expressed as a percentage of the total amount of phenolic compounds in each mixture, which ranged from 75 to 225 mg/100 g fresh weight of artificial diet. The lower value (75 mg) represents the rounded sum of the means of each compound contained in a number of apple cultivars including those showed to be resistant to *A. ludens* attack (Table 1) and was multiplied by three to reach the higher value (225 mg).

**Table 1:** The content of (+)-catechin, phloridzin, rutin, chlorogenic acid and *p*-coumaric acid in apple (*Malus* × *domestica*)

Compound	Content in *Malus* × *domestica* (mg/100 g FW)
(+)-Catechin	2.63 ± 0.84^a^
Phloridzin	5.87 ± 1.85^a^
Rutin	0.78 ± 0.35^a^
Chlorogenic acid	28.43 ± 9.04^a^
*p*-Coumaric acid	36.79 ± 3.1^b^

^a^Equals the mean of what was found in Grauer Hordaplfel, Engishofer, Bohnapfel, Schneiderapfel and Fuji apple cultivars, which were resistant to *Anastrepha ludens* attack (Aluja et al. 2014; J. Samietz pers. comm)

^b^Biedrzycka and Amarowicz 2008

Design points, involving combinations of phenolic compounds and concentrations, were selected using modified D-optimal criteria suitable for fitting a quadratic polynomial (Cornell 2002). The experiment included 45 model points, 5 lack-of-fit points, 45 replicated points, and 5 additional center points, for a total of 100 runs (Supplementary Table 2). The design had four block, 44 model, six lack of fit, and 45 pure error degrees of freedom. For logistic reasons, the experiment included five blocks to account for the number of treatments that could be performed at one time. The whole experiment (100 runs, Supplementary Table 2) was performed twice. The first experiment was run for 7 d, after which all larvae were recovered and weighed. The second experiment continued until adult emergence.

(+)-Catechin hydrate, rutin hydrate, and phloridzin dihydrate were dissolved in water before being mixed with diet, whereas chlorogenic and *p*-coumaric acids were anhydrous and were dissolved in 0.5 ml of 95 % ethanol prior to diet incorporation. A prior test for possible deleterious effects of 0.5 and 1 ml of 95 % ethanol in the response variables, analyzed by a one way *ANOVA*, found no significant effects (data not shown). As a result, ethanol was regarded as an inert solvent and was not considered further during analyses.

### Data Analyses

The measured responses at each design point were the mean values of all individuals found in Petri dishes. For each response variable, the highest order polynomial model in which additional model terms were significant and the lack of fit test non-significant (α = 0.05), was analyzed with an *ANOVA*. A series of adequacy tests as described by Anderson and Whitcomb (2005) were performed: normality and homoscedasticity were determined graphically *via* normal probability plots of residuals. Box-Cox plots were used to identify, if required, the necessity and type of data transformation. Overly influential data points were identified with DFFITS (a measure of influence based on the difference in fits in each predicted value) and DFBETAS (a measure of influence based on difference in model coefficients) plots (Belsley et al. 1980). The precision of the model was determined by comparing the range of the predicted values at the design points to the average variance of the prediction; potential outlier points were checked with externally studentized “*outlier-t*” (Weisberg 1985; Myers 1990) and Cook’s distance (Cook and Weisberg 1982) graphical plots. Multiple correlation coefficients (*R* ^2^, adjusted *R* ^2,^ and predicted *R* ^2^) were estimated for each selected model. The software Design-Expert ® 8 (Stat-Ease, Inc, Minneapolis, MN, USA) was used for experimental design construction, model evaluation, and all analyses.

## Results

A summary of the statistics for responses affected significantly by particular mixtures and concentrations of phenolic compounds is presented in Table 2. The diagnostics fell within acceptable limits (*i.e.*, results were normally distributed and displayed constant variance). With one exception, no outlier-*t* points were observed, no points exceeded a Cook’s distance of one, and predicted points were in close agreement with empirical values (data not shown). One point (run 37) in the larval weight experiment was identified as suspect by the outlier *t*-test and Cook’s distance analysis, and was therefore ignored during analysis. Larval and pupal weight, larval and pupal development time, pupation, emergence, survival (neonate to adult), and malformations of *A ludens* reared in artificial diet without added phenolic compounds were 19.2 (± 0.9) and 21.9 (± 0.3) mg, 9.9 (±0.1) and 13.7 (± 0.06) days, 83.9 (± 2.8) %, 95.8 (± 1.5) %, 82.7 (±3) %, and 2.4 (± 1.4) %, respectively (*N* = 12 Petri dishes each with 25 g of artificial diet and 30 *A. ludens* larvae).

**Table 2:** *ANOVA*, coefficient estimates, and summary statistics of developmental performance of *Anastrepha ludens* in response to different mixtures and amounts of (+)-catechin (Ca), phloridzin (Ph), rutin (Ru), chlorogenic acid (ChA), and *p*-coumaric acid (pCoA) added to its artificial diet

Effects	7 d old larval weight			Larval development time			Pupal development time			Adults emerged deformed		
	*F* Value	*P*-value	Coefficient estimate	*F* Value	*P*-value	Coefficient	*F* Value	*P*-value	Coefficient estimate	*F* Value	*P*-value	Coefficient estimate
Model	4.35	**<0.001**		5.55	**<0.001**		4.08	**<0.001**		3.88	**0.003**	
Linear mixture	2.26	>0.05		4.31	**0.003**		1.54	>0.05		1.13	>0.05	
Ca	–	–	21.11	–	–	10.60	–	–	15.06	–	–	0.62
Ph	–	–	18.82	–	–	10.62	–	–	15.18	–	–	0.78
Ru	–	–	18.71	–	–	10.68	–	–	14.89	–	–	0.75
ChA	–	–	21.09	–	–	10.79	–	–	15.01	–	–	0.83
pCoA	–	–	19.17	–	–	11.07	–	–	15.25	–	–	0.96
2-component/factor effects												
Ca × ChA	5.38	**0.023**	−10.07	–	–	–	–	–	–	–	–	–
Ca × Ru	3.66	>0.05	7.97	–	–	–	–	–	–	–	–	–
Ca × [Conc.]	5.05	**0.027**	1.76	–	–	–	–	–	–	14.89	**<0.001**	−0.50
ChA × pCoA	9.36	**0.003**	−12.68	–	–	–	–	–	–	–	–	–
Ph × Ru	12.39	**<0.001**	14.47	–	–	–	–	–	–	–	–	–
Ph × pCoA	–	–	–	11.57	**0.001**	−1.83	–	–	–	–	–	–
pCoA × [Conc.]	–	–	–	5.00	**0.028**	0.23	–	–	–	–	–	–
Ph × [Conc]	–	–	–	–	–	–	16.52	**<0.001**	0.66	–	–	–
3-component/factor effects												
Ca × ChA × [Conc.]	–	–	–	–	–	–	7.81	**0.006**	−2.04	–	–	–
Ca × Ph × [Conc.]	–	–	–	–	–	–	7.77	**0.007**	−2.32	–	–	–
ChA × Ru × [Conc.]	–	–	–	–	–	–	6.57	**0.012**	−1.86	–	–	–
ChA × pCoA × [Conc.]	–	–	–	–	–	–	2.91	>0.05	−1.25	–	–	–
Ph × Ru × [Conc.]	–	–	–	–	–	–	15.73	**<0.001**	−3.22	–	–	–
Ph × pCoA × [Conc.]	–	–	–	–	–	–	10.73	**0.001**	−2.67	–	–	–
Lack of fit	*P* = 0.551			*P* = 1			*P* = 0.885			*P* = 0.064		
Model type^a^	Reduced quadratic × linear			Reduced quadratic × linear			Reduced quadratic × linear			Reduced linear × linear		
Transformation^b^	None			None			None			Power (x + 1)^−2.6^		
*R* ^2^	0.31			0.27			0.35			0.18		
*R* ^2^_adj_	0.24			0.22			0.26			0.13		
*R* ^2^_pred_	0.08			0.09			0.10			0.0003		

Significant *P* - values (*P* < 0.05) appear in bold

^a^Model reduction by backward elimination

^b^Determined by a Box-Cox plot analysis

Combinations of particular mixtures and concentrations of phenolic compounds had no significant effects on pupal weight (*F* = 1.4; *df* = 6, 45; *P* = 0.2), percentage of pupation (*F* = 0.5; *df* = 4, 45; *P* = 0.7), percentage of adult emergence (*F* = 1.5; *df* = 5, 45; *P* = 0.2), or percentage of survival from neonate to adult (*F* = 0.6; *df* = 4, 45; *P* = 0.7).

### Larval Weight

**Fig. 1:**
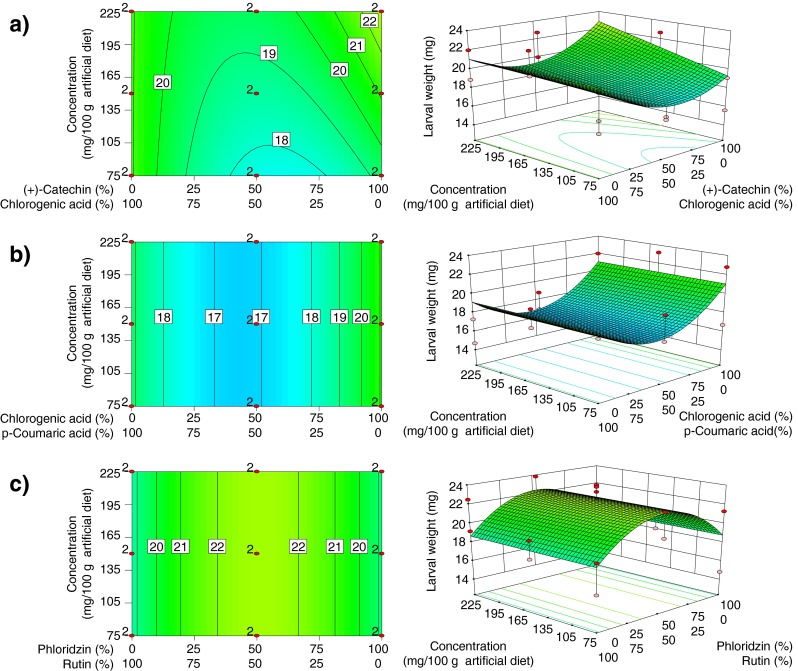
Response surface model showing significant model terms affecting larval weight (mg): **a** (+)-catechin × chlorogenic acid, and (+)-catechin × concentration; **b** chlorogenic acid × *p*-coumaric acid; and **c** phloridzin × rutin. Plots on the *left* indicate the proportional effects of mixture components along the *x*-axis and the concentration effect in mg/100 g of artificial diet along the *y*-axis. *Contour lines* indicate the response surface of larval weight. The plots on the *right* display the model in 3-D. Design points in *red* labeled “2” were replicated

Mean larval weight ranged from 14.4 – 26.5 mg. Fitting a reduced quadratic mixture × linear concentration model provided a highly significant explanation for observed response (*P* = 0.0001). Linear mixture was not significant (*P* = 0.07) indicating that larval weights did not vary significantly in the presence of single compounds (Table 2). The lack of fit test was not significant (*P* = 0.55) indicating that additional variation in the residuals could not be reduced by fitting a different model. The quadratic mixture × linear concentration model explained 24 % of the observed variance (*R* ^2^_adj_ = 0.24), with four model terms significantly affecting weight of larvae (Table 2). The model suggests that increasing the concentration of (+)-catechin in the diet resulted in heavier larvae, whereas mixtures of (+)-catechin and chlorogenic acid resulted in lower larval weights than those obtained when either of these compounds were present alone (Fig. 1a). Mixtures of chlorogenic and *p*-coumaric acids resulted in the lowest larval weights, independent of concentration (Fig. 1b), whereas phloridzin × rutin mixtures resulted in the highest weights (Fig. 1c).

### Larval Development Time

**Fig. 2:**
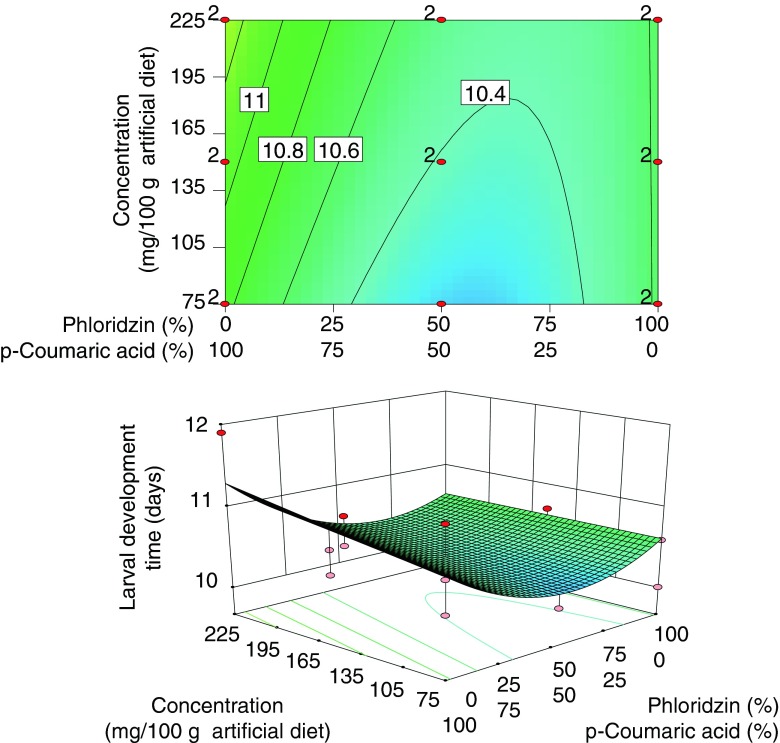
Response surface model showing significant model terms affecting larval development time. In the upper plot the proportional effects of mixture components are indicated along the *x*-axis and the concentration effect in mg/100 g of artificial diet along the *y*-axis. *Contour lines* indicate the response surface of larval development time. The lower plot displays the model in 3-D. Design points in *red* labeled “2” were replicated

Mean larval development time ranged from 9.9 – 11.9 days. A reduced quadratic mixture × linear concentration model was fitted (Table 2). The model was highly significant (*P* < 0.001), and the lack of fit test was not significant (*P* = 1). The model explained 22 % of the observed variance (*R* ^2^_adj_ = 0.22). The linear mixture, phloridzin × *p*-coumaric acid, and *p*-coumaric acid × (concentration) significantly affected larval development time as shown in the *ANOVA* model (Table 2). The model indicates that a high concentration of *p*-coumaric acid in the diet prolonged larval development time, whereas the mixture of phloridzin × *p*-coumaric acid resulted in shorter development times than those obtained when these compounds were present individually (Fig. 2).

### Pupal Development Time

**Fig. 3:**
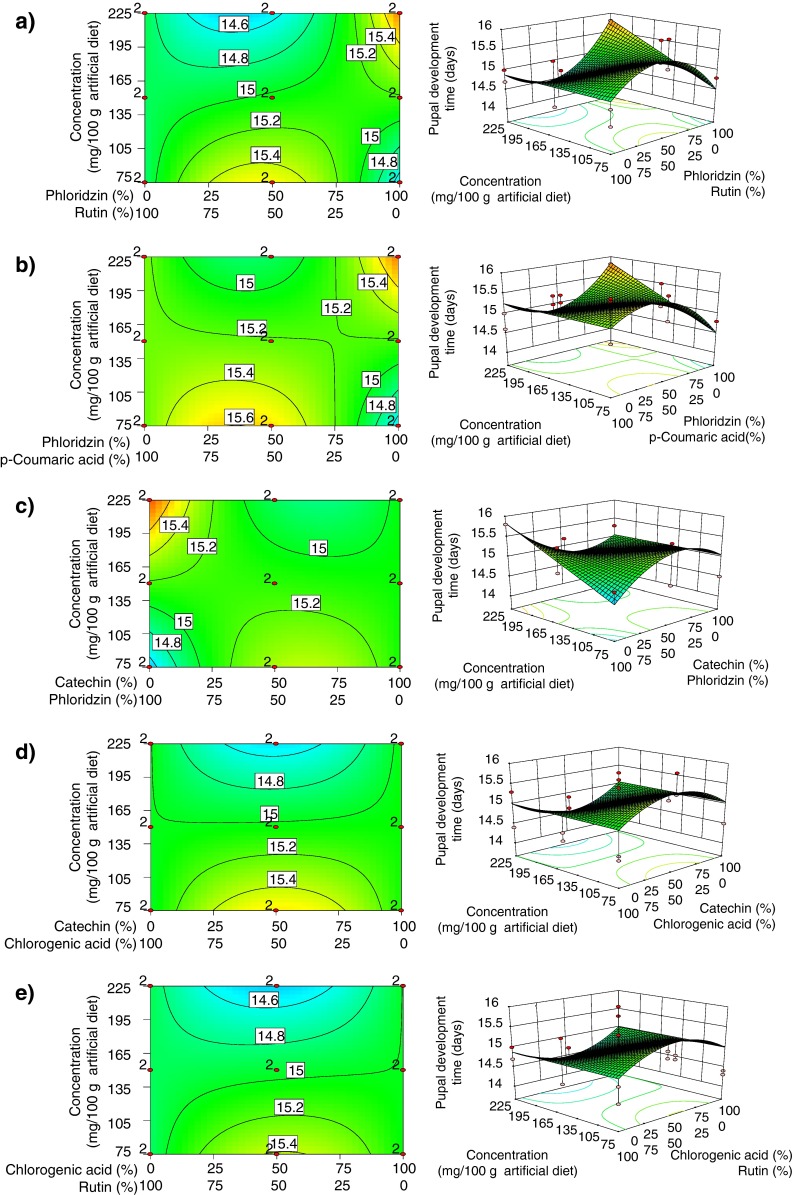
Response surface model showing significant model terms affecting pupal development time (days): **a** phloridzin × rutin × concentration; **b** phloridzin × *p*-coumaric acid × concentration; **c** (+)-catechin × phloridzin × concentration; **d** (+)-catechin × chlorogenic acid × concentration; and **e** chlorogenic acid × rutin × concentration. Plots on the *left* indicate the proportional effects of mixture components along the *x*-axis and the concentration effect in mg/100 g of artificial diet along the *y*-axis. *Contour lines* indicate the response surface of pupal development time. The plots on the *right* display the model in 3-D. Design points in red labeled “2” were replicated

Mean pupal development time ranged from 14.1 – 16.0 days, and the best fitting model was a reduced quadratic mixture × linear concentration. A lack of fit test was not significant (*P* = 0.88). The model was highly significant (*P* < 0.001), and explained 26 % of overall variation. *ANOVA* revealed six significant model terms (Table 2). Development time increased with the concentration of phloridzin, but an opposite tendency was observed in the phloridzin × rutin mixture, in which development times were >15.4 days at the lower concentrations and <14.6 days at the highest concentrations (Fig. 3a). A similar pattern was observed in the (+)-catechin × phloridzin, and the phloridzin × *p*-coumaric acid mixtures (Fig. 3c and e) with higher concentrations of (+)-catechin × chlorogenic acid, and chlorogenic acid × rutin resulting in shorter development times (Fig. 3b and d).

### Malformations in Adults

**Fig. 4:**
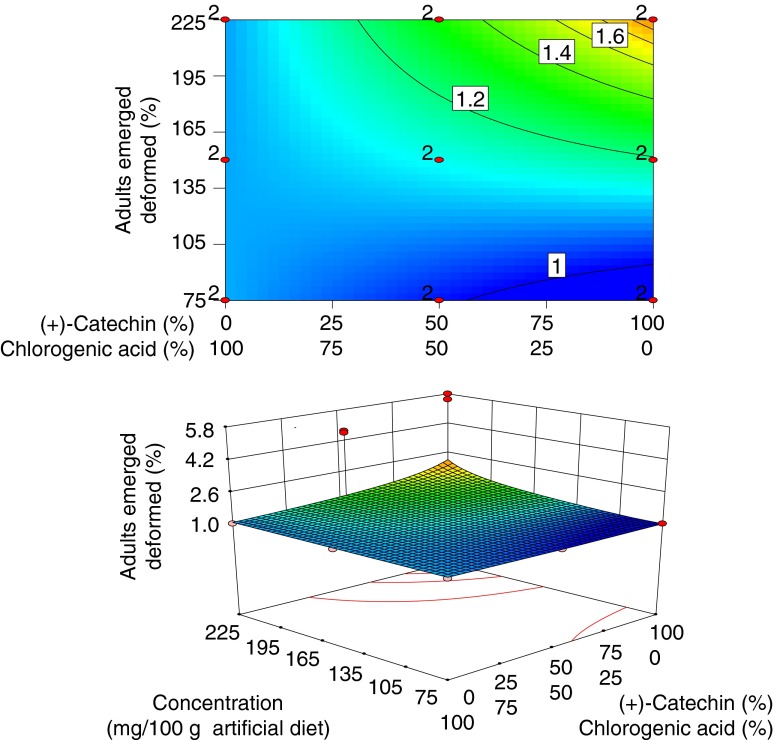
Response surface model showing significant model terms affecting percentage of adults that were deformed. In the *upper* plot the proportional effects of mixture components are indicated along the *x*-axis and the concentration effect in mg/100 g of artificial diet along the *y*-axis. *Contour lines* reflect the response surface of adults that were deformed. The *lower* plot displays the model in 3-D. Design points in *red* labeled “2” were replicated. Untransformed data are presented

The proportion of adults that were deformed ranged from 0 to 8 %. A reduced linear mixture × linear concentration model was selected (*P* = 0.003). Zero values were eliminated by (x + 1) transformation, and data were then normalized by power transformation as identified by Box-Cox plot analysis (Table 2). A lack of fit test was not significant (*P* = 0.06). The model explained 13 % of variation (*R* ^2^_adj_ = 0.13) and *ANOVA* indicated that adult malformations were significantly correlated only with high concentrations of (+)-catechin (Table 2, Fig. 4).

## Discussion

This is the first study that has employed a mixture-amount experimental design to examine the effect of phenolic compounds on the development of a phytophagous insect. Using this unique methodology, we observed that the effects of mixtures could not be predicted from the activities of their individual compounds. Furthermore, we discovered synergistic and antagonistic interactions among compounds of the same chemical class as well as among compounds of different classes. High concentrations of (+)-catechin resulted in significantly heavier larvae, but mixing this flavonoid with chlorogenic acid resulted in an antagonistic interaction as larval weights were reduced. Similarly, chlorogenic acid or *p*-coumaric acid did not significantly reduce larval weight but the opposite was observed when chlorogenic and *p*-coumaric acids were presented as a mixture. In contrast, the phloridzin and rutin mixture resulted in increased larval weight in a synergistic response. Larval development time was delayed as the concentration of *p*-coumaric acid increased, but this effect was counteracted by phloridzin, such that mixtures of phloridzin and *p*-coumaric acid resulted in an antagonistic effect with faster larval development than that observed with individual compounds. Pupal development time increased with increasing concentrations of phloridzin, although mixtures of phloridzin with rutin, (+)-catechin, or *p*-coumaric acid, and chlorogenic acid mixed with (+)-catechin or rutin resulted in an opposite trend. The data partially support our prediction that blends of phenolic compounds at high concentrations have more effect on insect development and survival than individual compounds at low concentrations. However, some compounds exhibited individual effects (*e.g.*, (+)-catechin) whereas others exhibited effects only when presented in mixtures.

One of the compounds we tested, chlorogenic acid, was previously reported to have no effect on larval development of the tephritid *Rhagoletis pomonella* (Walsh) (Pree 1977). In our study chlorogenic acid alone had no effect, but it affected larval weight when mixed with (+)-catechin or *p*-coumaric acid, and pupal development time when mixed with (+)-catechin or rutin. This confirms the usefulness of our experimental design for the simultaneous study of various compounds, and suggests that the effect of chlorogenic acid on tephritid development is conditioned by the presence of other compounds.

Larval development of *A. ludens* is slower in grapefruit (*Citrus paradisi* Macf.) or orange (*C. sinensis* Osbeck), than in peach (*Prunus persica* L) (Leyva et al. 1991). Both of the citrus species have *p*-coumaric acid in their pulp (Gorinstein et al. 2001), but peach does not (Andreotti et al. 2008; Blanda et al. 2008). Consistent with this previous report, we found that extended larval development times were correlated with dietary *p*-coumaric acid concentration.

Phenolic compounds can inhibit protein digestion in insect larvae as shown for the European spruce sawfly, *Gilpinia hercyniae* Htg., in which catechin restricted the gut protease activity, thus inhibiting digestion of dietary protein (Schopf 1986). Also, catechin was negatively correlated with protein content in pupal hemolymph in *A. ludens* (Aluja et al. 2014), suggesting an interaction among proteins and this compound. If (+)-catechin reduced protein digestion in *A. ludens* larvae in our study, then larvae may have compensated for nutritional deficiencies by increasing their rate of feeding, resulting in increased weight gains. A similar pattern was reported for locusts that responded to amino acid deficient diets by consuming significantly larger quantities of food (Simpson and Simpson 1990). Other studies on insects reared on artificial diets have suggested that heavier individuals have reduced survival rates (Lapointe et al. 2008). Therefore, heavier insect body weight may correlate with lower fitness. In our study high concentrations of (+)-catechin were associated with increased larval weights and an increase in the prevalence of malformed adults. The response surface in Fig. 4 suggests that further exploration of that space with higher concentrations might be useful.

Phenolic compounds including catechin, rutin, phloridzin, chlorogenic acid, epicatechin, procyanidin B1 and B2, coumaroylquinic acid, phloretin-xyloglucoside, and quercetin-glycosides in locally grown apple cultivars are correlated with increased mortality and lower pupal weights of *A. ludens* (Aluja et al. 2014) These authors also observed a negative relationship between catechin content and pupal weight. In contrast, we observed no significant effect with any mixture or concentration on pupal weightor on percentages of pupation, emergence, and survival, even at the highest concentrations with five-component mixtures. Moreover, (+)-catechin did not reduce pupal weight and was correlated with increased larval weight.

The contrasting results observed by Aluja et al. (2014) and the present study may be a consequence of the nutritional differences between the artificial diet that we used and the natural diet (host) of *A. ludens*. The laboratory diet contains 4–12 times the amount of protein observed in natural hosts, such as grapefruit or mango, and the protein: carbohydrate ratio of artificial diet (1: 3.8), grapefruit (1: 12.5) and mango (1: 27.8) are quite different (Cicero 2011). Adverse effects of secondary metabolites on the development of herbivorous insects are strongly correlated with protein content, and protein: carbohydrate ratios in the diet (Haukioja et al. 2002; Salvador et al. 2010; Simpson and Raubenheimer 2001). Therefore, the effects of these compounds on phytophagous insects may often be dampened by the high nutrient concentrations in artificial diets (Lapointe et al. 2008; Rose et al. 1988; Smith 2010).

More research on our study system is needed before we can propose a management strategy for the control of *A. ludens* based on manipulation of phenolic compounds in host fruit. For example, the interactions between phenolic compounds and nutrient levels, and the effects of mixtures on higher tropic levels such as parasitoids, have not been investigated. Nonetheless, we believe that some of the trends observed in our study suggest directions for future research. For example, prolonged larval and pupal development times of herbivore insects often lead to prolonged exposure to attack by natural enemies, thus increasing the mortality of individuals that develop slowly (Clancy and Price 1987; Rostas and Hilker 2003). By enhancing the accumulation of *p*-coumaric acid in the host fruits of trap cropping trees (Aluja and Rull 2009), larval development time of infesting flies might be increased and fly populations might suffer increased mortality caused by natural enemy attacks. Similarly, enhancing the levels of *p*-coumaric acid, chlorogenic acid or catechin in host fruit, or matching their proportions to nearly 50: 50 (%), could affect weight of infesting larvae as observed in the response surfaces of Fig. 2a, b.

Our study highlights the importance of testing not only the individual effects of potential defensive plant compounds, but also their combinations in order to understand how plants defend themselves against herbivores and how herbivores respond to plant defenses. When using artificial diets treated with secondary compounds, attention should be paid to the nutritional content of the diet. Ongoing studies are focused on mixture experimentation to modify the artificial diet of *A. ludens* and to test the hypothesis that the effects of phenolic compounds on this fly species are modulated by the nutritional content of the larval diet.

## Electronic supplementary material

Below is the link to the electronic supplementary material.

ESM 1 (DOCX 32 kb)
