# Supplementary material for: Mixture-Amount Design and Response Surface Modeling to Assess the Effects of Flavonoids and Phenolic Acids on Developmental Performance of Anastrepha ludens
Source: J Chem Ecol. 2014 Mar 12;40(3):297–306. doi: 10.1007/s10886-014-0404-6 (PMC3972442; doi:10.1007/s10886-014-0404-6)
Supplement: Supplementary file 1 — (DOCX 32 kb) [file 10886_2014_404_MOESM1_ESM.docx]

**SUPPLEMENTARY MATERIAL**

FOR: *Journal of Chemical Ecology*

A MIXTURE-AMOUNT DESIGN AND RESPONSE SURFACE MODELING TO ASSESS THE EFFECTS OF FLAVONOIDS AND PHENOLIC ACIDS ON DEVELOPMENTAL PERFORMANCE OF *Anastrepha ludens* (DIPTERA: TEPHRITIDAE)

CARLOS PASCACIO-VILLAFÁN^1*^, STEPHEN LAPOINTE^2^, TREVOR WILLIAMS^1^, JOHN SIVINSKI^3^, RANDALL NIEDZ^2^, and MARTÍN ALUJA^1^

^1^*Red de Manejo Biorracional de Plagas y Vectores, Instituto de Ecología, A.C. (INECOL), Xalapa, Veracruz, Mexico*

^2^*United States Horticultural Research Laboratory, Fort Pierce, Florida, U.S.A.*

^3^*Center for Medical, Agricultural and Veterinary Entomology, Gainesville, Florida, U.S.A.*

** Corresponding author - cpascacio@hotmail.com*

SUPPLEMENTARY TABLE 1 Chemical properties of the phenolic compounds tested

| Compound | Purity (%)^a^ | Chemical | Chemical | Empirical |
| --- | --- | --- | --- | --- |
|  |  | class^b^ | subclass^b^ | formula^a^ |
| (+)-Catechin | ≥ 98 | Flavonoid | Flavan-3-ol | C_15_H_14_O_6_ |
| Phloridzin | ≥ 99 | Flavonoid | Dihydrochalcone | C_21_H_24_O_10_ |
| Rutin | ≥ 94 | Flavonoid | Flavonol glycoside | C_27_H_30_O_16_ |
| Chlorogenic acid | ≥ 95 | Phenolic acid | Hydroxycinnamic ester | C_16_H_18_O_9_ |
| *p*-Coumaric acid | ≥ 98 | Phenolic acid | Hydroxycinnamic acid | C_9_H_8_O_3_ |

^a^ As informed by the supplier

^b^ Ribéreau-Gayon (1972), Vermerris and Nicholson (2008)

SUPPLEMENTARY TABLE 2 Mixture-amount design employed in a five block experiment

|  |  |  |  | Mixture components (%) | | | | | | | | |  | Factor |
| --- | --- | --- | --- | --- | --- | --- | --- | --- | --- | --- | --- | --- | --- | --- |
| Run |  | Block |  | Ca |  | Ph |  | Ru |  | ChA |  | pCoA |  | Concentration (mg/100 g diet) |
| 1 |  | Block 1 |  | 0 |  | 0 |  | 0 |  | 0 |  | 100 |  | 75 |
| 2 |  | Block 1 |  | 60 |  | 10 |  | 10 |  | 10 |  | 10 |  | 113 |
| 3 |  | Block 1 |  | 50 |  | 0 |  | 50 |  | 0 |  | 0 |  | 225 |
| 4 |  | Block 1 |  | 0 |  | 0 |  | 50 |  | 0 |  | 50 |  | 225 |
| 5 |  | Block 1 |  | 0 |  | 0 |  | 50 |  | 0 |  | 50 |  | 150 |
| 6 |  | Block 1 |  | 20 |  | 20 |  | 20 |  | 20 |  | 20 |  | 150 |
| 7 |  | Block 1 |  | 50 |  | 50 |  | 0 |  | 0 |  | 0 |  | 225 |
| 8 |  | Block 1 |  | 0 |  | 0 |  | 50 |  | 0 |  | 50 |  | 225 |
| 9 |  | Block 1 |  | 0 |  | 100 |  | 0 |  | 0 |  | 0 |  | 75 |
| 10 |  | Block 1 |  | 0 |  | 0 |  | 50 |  | 50 |  | 0 |  | 225 |
| 11 |  | Block 1 |  | 50 |  | 0 |  | 50 |  | 0 |  | 0 |  | 225 |
| 12 |  | Block 1 |  | 50 |  | 0 |  | 0 |  | 50 |  | 0 |  | 225 |
| 13 |  | Block 1 |  | 50 |  | 50 |  | 0 |  | 0 |  | 0 |  | 225 |
| 14 |  | Block 1 |  | 0 |  | 50 |  | 50 |  | 0 |  | 0 |  | 225 |
| 15 |  | Block 1 |  | 0 |  | 0 |  | 0 |  | 0 |  | 100 |  | 75 |
| 16 |  | Block 1 |  | 10 |  | 10 |  | 60 |  | 10 |  | 10 |  | 113 |
| 17 |  | Block 1 |  | 0 |  | 100 |  | 0 |  | 0 |  | 0 |  | 75 |
| 18 |  | Block 1 |  | 0 |  | 0 |  | 50 |  | 50 |  | 0 |  | 225 |
| 19 |  | Block 1 |  | 50 |  | 0 |  | 0 |  | 50 |  | 0 |  | 225 |
| 20 |  | Block 1 |  | 0 |  | 50 |  | 50 |  | 0 |  | 0 |  | 225 |
| 21 |  | Block 2 |  | 0 |  | 50 |  | 0 |  | 0 |  | 50 |  | 75 |
| 22 |  | Block 2 |  | 50 |  | 50 |  | 0 |  | 0 |  | 0 |  | 75 |
| 23 |  | Block 2 |  | 0 |  | 0 |  | 0 |  | 50 |  | 50 |  | 75 |
| 24 |  | Block 2 |  | 10 |  | 10 |  | 10 |  | 10 |  | 60 |  | 188 |
| 25 |  | Block 2 |  | 0 |  | 100 |  | 0 |  | 0 |  | 0 |  | 150 |
| 26 |  | Block 2 |  | 50 |  | 0 |  | 0 |  | 0 |  | 50 |  | 75 |
| 27 |  | Block 2 |  | 0 |  | 100 |  | 0 |  | 0 |  | 0 |  | 150 |
| 28 |  | Block 2 |  | 0 |  | 100 |  | 0 |  | 0 |  | 0 |  | 225 |
| 29 |  | Block 2 |  | 20 |  | 20 |  | 20 |  | 20 |  | 20 |  | 150 |
| 30 |  | Block 2 |  | 50 |  | 50 |  | 0 |  | 0 |  | 0 |  | 75 |
| 31 |  | Block 2 |  | 0 |  | 0 |  | 0 |  | 100 |  | 0 |  | 150 |
| 32 |  | Block 2 |  | 0 |  | 0 |  | 100 |  | 0 |  | 0 |  | 225 |
| 33 |  | Block 2 |  | 50 |  | 0 |  | 0 |  | 0 |  | 50 |  | 75 |
| 34 |  | Block 2 |  | 0 |  | 0 |  | 50 |  | 0 |  | 50 |  | 75 |
| 35 |  | Block 2 |  | 0 |  | 100 |  | 0 |  | 0 |  | 0 |  | 225 |
| 36 |  | Block 2 |  | 0 |  | 0 |  | 0 |  | 50 |  | 50 |  | 75 |
| 37 |  | Block 2 |  | 0 |  | 50 |  | 0 |  | 0 |  | 50 |  | 75 |
| 38 |  | Block 2 |  | 0 |  | 0 |  | 100 |  | 0 |  | 0 |  | 225 |
| 39 |  | Block 2 |  | 0 |  | 0 |  | 50 |  | 0 |  | 50 |  | 75 |
| 40 |  | Block 2 |  | 0 |  | 0 |  | 0 |  | 100 |  | 0 |  | 150 |
| 41 |  | Block 3 |  | 20 |  | 20 |  | 20 |  | 20 |  | 20 |  | 150 |
| 42 |  | Block 3 |  | 0 |  | 50 |  | 0 |  | 0 |  | 50 |  | 225 |
| 43 |  | Block 3 |  | 0 |  | 50 |  | 0 |  | 50 |  | 0 |  | 75 |
| 44 |  | Block 3 |  | 0 |  | 0 |  | 0 |  | 50 |  | 50 |  | 225 |
| 45 |  | Block 3 |  | 0 |  | 0 |  | 100 |  | 0 |  | 0 |  | 150 |
| 46 |  | Block 3 |  | 100 |  | 0 |  | 0 |  | 0 |  | 0 |  | 150 |
| 47 |  | Block 3 |  | 0 |  | 50 |  | 0 |  | 0 |  | 50 |  | 225 |
| 48 |  | Block 3 |  | 100 |  | 0 |  | 0 |  | 0 |  | 0 |  | 150 |
| 49 |  | Block 3 |  | 50 |  | 0 |  | 50 |  | 0 |  | 0 |  | 75 |
| 50 |  | Block 3 |  | 0 |  | 50 |  | 0 |  | 50 |  | 0 |  | 75 |
| 51 |  | Block 3 |  | 0 |  | 0 |  | 0 |  | 50 |  | 50 |  | 225 |
| 52 |  | Block 3 |  | 0 |  | 50 |  | 50 |  | 0 |  | 0 |  | 75 |
| 53 |  | Block 3 |  | 0 |  | 50 |  | 0 |  | 50 |  | 0 |  | 225 |
| 54 |  | Block 3 |  | 0 |  | 0 |  | 0 |  | 0 |  | 100 |  | 150 |
| 55 |  | Block 3 |  | 0 |  | 50 |  | 0 |  | 50 |  | 0 |  | 225 |
| 56 |  | Block 3 |  | 0 |  | 0 |  | 0 |  | 0 |  | 100 |  | 150 |
| 57 |  | Block 3 |  | 0 |  | 50 |  | 50 |  | 0 |  | 0 |  | 75 |
| 58 |  | Block 3 |  | 20 |  | 20 |  | 20 |  | 20 |  | 20 |  | 150 |
| 59 |  | Block 3 |  | 0 |  | 0 |  | 100 |  | 0 |  | 0 |  | 150 |
| 60 |  | Block 3 |  | 50 |  | 0 |  | 50 |  | 0 |  | 0 |  | 75 |
| 61 |  | Block 4 |  | 50 |  | 0 |  | 50 |  | 0 |  | 0 |  | 150 |
| 62 |  | Block 4 |  | 50 |  | 0 |  | 0 |  | 0 |  | 50 |  | 150 |
| 63 |  | Block 4 |  | 0 |  | 0 |  | 50 |  | 50 |  | 0 |  | 150 |
| 64 |  | Block 4 |  | 50 |  | 50 |  | 0 |  | 0 |  | 0 |  | 150 |
| 65 |  | Block 4 |  | 0 |  | 50 |  | 0 |  | 0 |  | 50 |  | 150 |
| 66 |  | Block 4 |  | 50 |  | 0 |  | 50 |  | 0 |  | 0 |  | 150 |
| 67 |  | Block 4 |  | 50 |  | 50 |  | 0 |  | 0 |  | 0 |  | 150 |
| 68 |  | Block 4 |  | 0 |  | 50 |  | 50 |  | 0 |  | 0 |  | 150 |
| 69 |  | Block 4 |  | 0 |  | 50 |  | 50 |  | 0 |  | 0 |  | 150 |
| 70 |  | Block 4 |  | 50 |  | 0 |  | 0 |  | 50 |  | 0 |  | 150 |
| 71 |  | Block 4 |  | 0 |  | 50 |  | 0 |  | 50 |  | 0 |  | 150 |
| 72 |  | Block 4 |  | 0 |  | 50 |  | 0 |  | 50 |  | 0 |  | 150 |
| 73 |  | Block 4 |  | 0 |  | 50 |  | 0 |  | 0 |  | 50 |  | 150 |
| 74 |  | Block 4 |  | 50 |  | 0 |  | 0 |  | 0 |  | 50 |  | 150 |
| 75 |  | Block 4 |  | 0 |  | 0 |  | 50 |  | 50 |  | 0 |  | 150 |
| 76 |  | Block 4 |  | 50 |  | 0 |  | 0 |  | 50 |  | 0 |  | 75 |
| 77 |  | Block 4 |  | 0 |  | 0 |  | 0 |  | 50 |  | 50 |  | 150 |
| 78 |  | Block 4 |  | 50 |  | 0 |  | 0 |  | 50 |  | 0 |  | 75 |
| 79 |  | Block 4 |  | 0 |  | 0 |  | 0 |  | 50 |  | 50 |  | 150 |
| 80 |  | Block 4 |  | 50 |  | 0 |  | 0 |  | 50 |  | 0 |  | 150 |
| 81 |  | Block 5 |  | 100 |  | 0 |  | 0 |  | 0 |  | 0 |  | 225 |
| 82 |  | Block 5 |  | 0 |  | 0 |  | 100 |  | 0 |  | 0 |  | 75 |
| 83 |  | Block 5 |  | 100 |  | 0 |  | 0 |  | 0 |  | 0 |  | 75 |
| 84 |  | Block 5 |  | 0 |  | 0 |  | 50 |  | 50 |  | 0 |  | 75 |
| 85 |  | Block 5 |  | 0 |  | 0 |  | 0 |  | 0 |  | 100 |  | 225 |
| 86 |  | Block 5 |  | 50 |  | 0 |  | 0 |  | 0 |  | 50 |  | 225 |
| 87 |  | Block 5 |  | 10 |  | 10 |  | 60 |  | 10 |  | 10 |  | 188 |
| 88 |  | Block 5 |  | 100 |  | 0 |  | 0 |  | 0 |  | 0 |  | 225 |
| 89 |  | Block 5 |  | 100 |  | 0 |  | 0 |  | 0 |  | 0 |  | 75 |
| 90 |  | Block 5 |  | 0 |  | 0 |  | 0 |  | 100 |  | 0 |  | 75 |
| 91 |  | Block 5 |  | 20 |  | 20 |  | 20 |  | 20 |  | 20 |  | 150 |
| 92 |  | Block 5 |  | 0 |  | 0 |  | 0 |  | 100 |  | 0 |  | 75 |
| 93 |  | Block 5 |  | 0 |  | 0 |  | 0 |  | 100 |  | 0 |  | 225 |
| 94 |  | Block 5 |  | 50 |  | 0 |  | 0 |  | 0 |  | 50 |  | 225 |
| 95 |  | Block 5 |  | 0 |  | 0 |  | 0 |  | 100 |  | 0 |  | 225 |
| 96 |  | Block 5 |  | 0 |  | 0 |  | 50 |  | 50 |  | 0 |  | 75 |
| 97 |  | Block 5 |  | 0 |  | 0 |  | 0 |  | 0 |  | 100 |  | 225 |
| 98 |  | Block 5 |  | 10 |  | 10 |  | 10 |  | 10 |  | 60 |  | 113 |
| 99 |  | Block 5 |  | 0 |  | 0 |  | 50 |  | 0 |  | 50 |  | 150 |
| 100 |  | Block 5 |  | 0 |  | 0 |  | 100 |  | 0 |  | 0 |  | 75 |

Experiment was a five-component mixture of the flavonoids (+)-catechin (Ca), phloridzin (Ph) and rutin (Ru), and the phenolic acids chlorogenic acid (ChA) and *p*-coumaric acid (pCoA), and one quantitative factor (total concentration of phenolic mixture/100 g of diet). The percentage of each component in the mixture is shown with the total concentration of phenolics in the experimental diet.
